# Optimal Enteral Nutrition Support Preserved Muscle Mass in Critically Ill Children

**DOI:** 10.1155/2022/7004543

**Published:** 2022-01-25

**Authors:** Kantisa Sirianansopa, Chavisa Rassameehirun, Sirinuch Chomtho, Orapa Suteerojntrakool, Lalida Kongkiattikul

**Affiliations:** ^1^Division of Pulmonology and Critical Care, Department of Pediatrics, Faculty of Medicine, Chulalongkorn University, Bangkok, Thailand; ^2^Division of Nutrition, Department of Pediatrics, Faculty of Medicine, Chulalongkorn University, Bangkok, Thailand; ^3^Pediatric Nutrition Research Unit, Department of Pediatrics, Faculty of Medicine, Chulalongkorn University, Bangkok, Thailand; ^4^Division of Ambulatory, Department of Pediatrics, Faculty of Medicine, Chulalongkorn University, Bangkok, Thailand

## Abstract

**Background:**

Inflammation and immobility are the most relevant mechanisms that alter protein synthesis and increase protein breakdown. Protein catabolism is associated with morbidity and mortality in critically ill children.

**Objective:**

To demonstrate the effectiveness of the routinely used enteral nutrition support guideline in preventing muscle breakdown in critically ill children.

**Methods:**

A prospective cohort study was conducted in the pediatric intensive care unit (PICU) of a tertiary care hospital. Critically ill children (aged 1 month to 15 years) admitted to the PICU were enrolled. All patients were assessed for nutritional status and nutritional requirement. Enteral nutrition support following the guideline was initiated within the first 24 hours if no contraindication. The calorie target was defined either by direct measurement from indirect calorimetry or estimated from Schofield equation with protein target at least 1.5 g/kg/day. Anthropometric assessments and body composition measurements by bioelectrical impedance analysis (BIA) were examined at baseline and on the seventh day of the PICU admission.

**Results:**

Sixty-three patients were enrolled in the study. The most common age group was 1–5 years old (38.1%). The length of PICU stay was 9.1 (SD = 12.7) days. Respiratory problems were the major cause of PICU admission (50.8%). Mechanical ventilation was required in 55.6% of the patients with the average duration of 6.3 (SD = 12.4) days. Undernutrition was found in 36.5% of the patients. Enteral feeding was the major route of nutrition support (95.2%). After the first week of admission, muscle mass was significantly preserved (*p* < 0.01). All patients received the nutrition support at their target energy and protein goal within the first week. The enteral feeding-related complication was reported in 1.6% of the patients.

**Conclusion:**

Protein catabolism during critically ill period can be minimized by optimal nutrition support. Nutrition practice using the enteral nutrition support guideline was effective in helping critically ill children reach their target caloric and protein intake.

## 1. Introduction

The metabolic response induced by critical illness activates catabolic pathways and causes resistance to anabolic signals, which leads to extensive muscle wasting and loss of muscle function [1]. Catabolism of body protein due to starvation, immobility, stress, and inflammation has been described in pediatric patients with critical illness. Patients in pediatric intensive care unit (PICU) are at a high risk of developing low or depleted protein reserves. In addition, most of these patients already have chronic illness with associated malnutrition. These conditions will increase their morbidity and mortality risks [2]. Protein depletion (15–25% of the total muscle mass) was reported during the first 10 days following admission to an intensive care unit [3]. Negative protein balance may result in loss of muscle mass which is associated with an increased length of hospital stay [4].

Therefore, prevention of muscle breakdown in the first week of the critically ill period is a challenging nutrition goal in critical care. Various modalities of body composition measurement can be used in order to monitor the muscle mass of critically ill patients [5]. Bioelectrical impedance analysis (BIA), a method for estimating body composition, was used in this study. The advantages of BIA are that it is noninvasive, there is no radiation exposure, and it is a simple procedure that can be performed at bedside in the supine position. Body components were classified by the BIA technique, including conductive fluid, nonconductive fluid, and tissue components. Moreover, the accuracy of muscle mass measurements is not influenced by the hydration status of the patients [6]. Body cell mass (BCM) is a BIA parameter and represents the protein rich compartment of the human body which is affected by catabolic stages. Loss of BCM is associated with poor clinical outcome [7]. Another BIA-derived parameter is phase angle. It was a correlation between the resistance and reactance vectors which indicated the cell membrane integrity and health fitness. PA has been studied as a prognostic marker in several health conditions including critically ill patients [8, 9].

Adequate nutrition support during the critically ill period is equally important to the recovery phase or rehabilitation period. Nutrition support focuses on reaching the energy goal with adequate micro- and macronutrients supplements. A lack of uniform bedside management tools could impact the nutrition outcome. Recently, the American Society for Parenteral and Enteral Nutrition (ASPEN) released a guideline for critically ill patients to achieve optimal nutrition support [10]. The previous nutrition practice guidelines in our institution were based on observational or retrospective data. To integrate with the update knowledge from the literature and the new ASPEN guideline, we developed an updated enteral nutrition support guideline for our own practice. A straightforward enteral nutrition support guideline consisted of five main concepts: (1) nutritional status and growth assessment, (2) nutritional requirement, (3) enteral nutrition advancement methods, (4) definition of gastrointestinal intolerance and stepwise approach management, and (5) bowel management strategies.

This prospective study aimed to evaluate the effectiveness of our enteral nutrition support guideline. We hypothesized that (1) the protocol complying with the guideline would result in optimal nutrition support and consequently prevent muscle breakdown in critically ill children, and (2) optimizing nutrition therapy was a potential avenue of improving clinical outcomes in critically ill pediatric patients.

## 2. Materials and Methods

### 2.1. Study Design and Setting

This prospective cohort study was conducted at the pediatric intensive care unit, the King Chulalongkorn Memorial Hospital, a university-affiliated hospital. This 10-bed PICU was a mixed medical-surgical unit providing medical service at the tertiary care level. This study was approved by the Institutional Review Board, Faculty of Medicine, Chulalongkorn University, IRB No. 401/63.

### 2.2. Study Population

From September 2020 to February 2021, all critically ill children aged from 1 month to 15 years admitted to the PICU at least 48 hours were included in the study. Exclusion criteria were children with a contraindication to enteral feeding (e.g., postoperative gastrointestinal tract surgery) or unable to obtain body composition assessment by BIA (e.g., limb amputation).

### 2.3. Sample Size

Sample size was calculated to detect a difference between the two means of dependent samples, with *z* = 1.96 or 95% confidence interval (CI) and bases on a standard deviation (SD) of 15 and the difference between the two groups of 6.3, reported by Hejazi et al. [11]. The calculated sample size of this study was 45.

### 2.4. Data Collection

Baseline characteristics were recorded which included age, sex, admission diagnosis, length of PICU stay, length of mechanical ventilation, and the Pediatric Risk of Mortality PRISM III score. Nutrition status (determined by anthropometric assessments and body composition assessments) was assessed within 48 hours after PICU admission and on the 7^th^ day of admission. The pediatrics resident who was in charge of each patient was instructed to follow the enteral nutrition support guideline (Supplement 1). Primary outcome was changed from baseline in the quantity of muscle mass at the end of first week in the PICU. Secondary outcomes were impacts of nutrition practice on clinical outcomes.

Anthropometric assessments were performed by well-trained pediatric residents. The measurements included weight, length or height, head circumference (only for children aged <36 months), mid-upper arm circumference (MUAC), and waist and hip circumference. For critically ill children who could not be weighed by a scale (e.g., mechanical ventilation or sedation), the weight was estimated by length/height measurement using the reference values from the World Health Organization (WHO) and nutrition status was classified by MUAC [12]. The anthropometric measurements categorized these patients in three main groups: normal nutrition status, protein energy malnutrition (weight for length/height < −3 *Z*-score), and obesity (weight for length/height > +3 *Z*-score) [13].

*Body composition* was measured by using BIA [7] (Bodystat®: Quadscan 4000, U.K.). Four electrodes were connected to the right hand (wrist and middle fingers) and the right foot (ankle and above the knuckle of the toe) in the supine position. The BIA analysis took approximately 2 minutes to measure the whole-body compositions. The body compositions were examined twice at baseline and on the seventh day of admission in the morning. The BIA was performed by the same technician who is regularly using this instrument. BIA estimated the body components which included fat free mass (FFM), total body water (TBW), and body cell mass (BCM). All of the body components except fat is classified as FFM. BCM, a part of FFM, was a protein rich compartment without extracellular water (ECW) and associated with catabolic states. Phase angle (PA) was calculated from the values obtained from BIA and has been studied as a prognostic marker in several health conditions [10].

Energy expenditure was assessed by indirect calorimetry (IC) (CARESCAPE™ R860, U.S.) in all mechanically ventilated patients [14]. If indirect calorimetry was not available or the patients were not intubated, an estimated resting energy expenditure (REE) was calculated from the Schofield equation without additional stress factors [15]. Our protocol did not calculate protein requirement but provided minimal protein intake at least 1.5 g/kg/day.

### 2.5. Statistical Analysis

All statistical analyses were performed using the R software, version 4.0.4 (Free Software Foundation, Inc., Boston, USA). Categorical variables were presented as frequency and percentage. Continuous variables were presented as mean with SD or median with interquartile range (IQR). The Wilcoxon signed-rank test was used to compare the mean difference (MD) of continuous data. The Spearmen's rank correlation test was used to assess relationships between the phase angle and mortality. A *p* value less than 0.05 indicated statistical significance.

## 3. Results

There were 64 critically ill children admitted in the PICU during the study period, of which 63 were enrolled in this cohort study. One patient was excluded due to incomplete follow-up on body composition on the 7^th^ day from extensive severe skin infection. All of the patients compiled with the enteral nutrition support by the guideline. The patient characteristics are described in Table 1. The reasons for admission were medical problems (79.3%), elective surgery (15.5%), and trauma/burn (3.2%). Medical problems included respiratory diseases (50.8%), cardiovascular diseases (14.3%), endocrine diseases (7.9%), and neurological diseases (6.3%). Energy expenditure was calculated by the Schofield equation, for REE (73% of the patients) and by indirect calorimetry (27%). Almost all of the patients (95.2%) received nutrition by enteral route.

**Figure 1:**
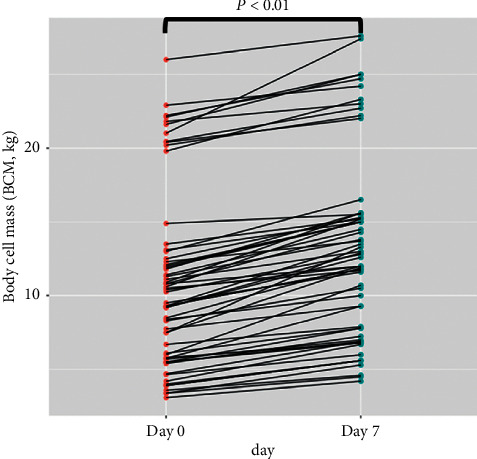
Demonstration of body cell mass, measured by BIA, on Day 7 of PICU admission. The BCM on Day 7 was not decreased compared with the first day of PICU admission (Wilcoxon signed-rank test, *p* < 0.01). BCM, body cell mass; BIA, bioelectrical impedance analysis; PICU, pediatric intensive care unit.

Muscle mass was measured by BIA and represented by BCM. The BCM after the first week of PICU admission was not decreased from the baseline (*p* < 0.01), as shown in Figure 1. Mean difference and SD of BCM were 2.9 and 2.4 kg. The PA was significantly increased after the first week of PICU admission (MD 2.0, 95% CI 1.5–2.4). Furthermore, an association between the PA and the PRISM III score was observed. The lower PA value tended to correlate with higher PRISM III score (*R* = −0.1, 95% CI −0.34, 0.15).

The nutritional and clinical outcomes are described in Table 2. All of the patients achieved their target nutrition support, in both calories and protein, by the end of the first week. A majority of the patients (81%) received enteral feeding within 48 hours of PICU admission. Feeding intolerance was reported in 13 patients (20.6%), of which 12 were improved after conservative management. Three patients (4.8%) had nonserious adverse events including enteral-related complication (necrotizing enterocolitis, 1.6%) and parenteral-related complication (catheter-related infection, 3.2%). Hospital-acquired infections were found in 27% of the patients, including catheter-related blood stream infection (CRBSI, 14.3%), Foley catheter-associated urinary tract infection (FA-UTI, 1.6%), nosocomial diarrhea (3.2%), and ventilator associated pneumonia (VAP, 7.9%).

**Figure 2:**
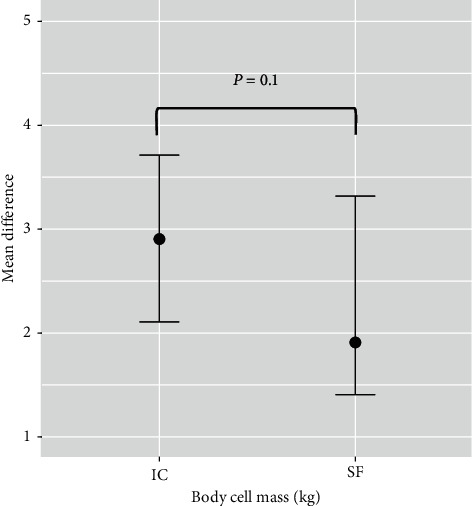
Comparison of mean differences of body cell mass between indirect calorimetry (IC) and Schofield equation (SF). Using either IC or the Schofield equation to estimate the target caloric goal could preserve muscle mass during PICU admission (Wilcoxon signed-rank test, *p*=0.01).

Subgroup analysis was performed to evaluate the treatment effect between the two different energy expenditure (the Schofield equation and the IC) calculation methods. The mean difference of muscle mass in the IC group was 2.9 (95% CI 2.1–3.7) and the Schofield equation group was 1.9 (95% CI 1.4–3.3), respectively (Figure 2). There was no statistical difference between the groups (*p*=0.01).

Factors associated with feeding intolerance were delayed time (>48 hours after admission) to start enteral feeding (adjusted OR 1.4, 95% CI 0.18–10.95) and undernutritional status before admission (adjusted OR 1.1, 95% CI 0.16–7.02).

## 4. Discussion

Optimal nutrition therapy in the critically ill period is associated with lower odds of 60 d mortality [2]. Nutrition support in critically ill children may be challenging because acute illness can alter the patient's metabolism which causes difficulty in anticipating the individual calorie needs. Moreover, many factors, such as a lack of uniform feeding approach at the bedside, can interrupt nutrient delivery in PICUs. Many intensive care units developed their own nutrition support guideline following the ASPEN recommendation [10]. The guidelines aim to achieve the target calories and protein intake with an ultimate goal of improving both short-term and long-term clinical outcomes. Decreased muscle mass was reported during critical illness and was related to worse outcomes [11]. BIA is a method used for measuring body composition which included muscle mass [16]. This study measured BCM to represent the muscle mass in the patients who received enteral nutrition support following the guideline. The results demonstrated that the BCM was not significantly decreased after a week of PICU admission.

We postulated that a nutrition support guideline could help critically ill children reach their target calories and protein intake. Our population from this study may be slightly different from usual due to COVID-19 pandemic as we did not have many severe critically ill patients. In contrast to previous studies, our patients achieved their caloric and protein target at the end of first week with a higher rate [17, 18]. Optimal nutrition management could help prevent muscle from breaking down. Minimal protein provided in our protocol was 1.5 g/kg/day; however, all patients in our study received at least 2 g/kg/day of protein intake at the end of first week. The important strategy of our guideline was aiming positive nitrogen balance which might be essential to offset the catabolic loss in critically ill children. Nevertheless, this study did not measure nitrogen balance which warrants further study. Weijs et al. [19] suggested at least 1.2 g/kg/day of protein intake for prevention of protein catabolism in the critically ill period. Our enteral nutrition support guideline was effective to minimize protein catabolism in the first week of critically ill period.

Provision of adequate energy intake for critically ill children is associated with the calculation of REE. IC is the goal standard to estimate REE. If it is not feasible, the Schofield equation is an alternative method for calculating daily energy requirement [14]. The results of this study suggested that both IC and the Schofield equation provided the same primary outcome that the muscle mass was preserved during PICU admission. Therefore, daily energy expenditure can be prescribed by using either method. Reaching the energy target was a potential goal; all patients in this study achieved their energy goal by the first week of PICU admission. Mehta et al. [20] revealed that an increase in energy intake from 33% to 66% of the prescription goal was associated with lower mortality (OR 0.27, 95% CI 0.11–0.67, *p*=0.002). The considerable obstacles were feeding intolerance symptoms. A stepwise approach was recommended as a bedside management tool for physicians to overcome these nutrition barriers. There were nonserious adverse events that occurred in our study period. The problem related to enteral feeding complication was observed only in 1.6% of the patients. Septic ileus occurred after encouraged bolus enteral feeding but was improved after conservative management.

PA is another BIA-derived parameter besides the BCM. PA is associated with mortality outcomes and has stronger predictive power of mortality than the severity scoring systems commonly used in many intensive care units (OR 0.53, *p* < 0.01) [21]. Recently, Zamberlan et al. [22] showed a strong correlation of PA > 2.8^◦^ with lower mortality rate in sepsis children (*p* < 0.01). This study found a negative correlation between the PA and PRISM III score (*r* = −0.1, 95% CI −0.34–0.15). A lower value of PA was associated with a higher severity score. Although this study was not designed to have enough power to detect this correlation, the trend of negative correlation was similar to another study [23]. PA could be used as an additional noninvasive indicator to predict prognosis in critically ill children.

To our knowledge, this is the first study that demonstrates the efficacy of enteral nutrition support guideline by using BIA to monitor muscle mass in critically ill children. Limitations of this study included being conducted during the coronavirus pandemic period, and therefore, the number of our cases in PICU was limited, especially the elective surgery cases. The BIA was measured in the morning regardless of fasting status; however, this was measured by the same person throughout the study period. The schedule to follow up the patient after PICU discharge was canceled due to the pandemic. There was no mortality in this study; therefore, we could not conclude this clinical outcome. There was no patient who received only parenteral nutrition during the study period; thus, we could not illustrate the effectiveness of our nutrition guideline on this group. Further study is required to evaluate in other groups of critically ill children, especially one with extracorporeal organ support, and the long-term impact of nutrition management in critically ill children.

## 5. Conclusion

Appropriate nutrition support during PICU admission plays a crucial role in the vital outcomes. Nutrition support guideline can be used for nutritional management in critically ill children which helps minimize protein catabolism. Nutrition practice using the enteral nutrition support guideline was effective in helping critically ill children reach their target caloric and protein intake.

## Figures and Tables

**Table 1:** Patient's characteristics of participating critically ill children (*N* = 63).

Characteristics	Mean (SD) or *N* (%)
Age (year)	
<1	18 (28.6)
1–5	24 (38.1)
6–10	11 (17.5)
11–15	10 (15.9)
Gender	
Men	30 (47.6)
Admission categories	
Medical	50 (79.3)
Surgical (elective)	11 (17.5)
Trauma/burn	2 (3.2)
PICU^†^ stay (day)	9.1 (12.7)
Respiratory support	
Mechanical ventilation	35 (55.6)
Ventilator (day)	6.3 (12.4)
Nutritional status assessment within 48 h	63 (100)
Normal	35 (55.6)
Mild PEM^††^	12 (19)
Moderate PEM	8 (12.7)
Severe PEM	3 (4.8)
Obesity	5 (7.9)
Energy expenditure prescription methods	
Schofield equation	46 (73)
Indirect calorimetry	17 (27)
Sources of nutrition	
Enteral nutrition only	60 (95.2)
Both enteral and parenteral	3 (4.8)
PRISM^†††^ III score (median^‡^, IQR)	5 (0, 11.5)

^†^PICU, pediatric intensive care unit;^††^PEM, protein energy malnutrition;^†††^PRISM, pediatric risk of mortality; ^‡^IQR, interquartile range.

**Table 2:** Clinical and nutritional outcomes.

Clinical and nutritional outcomes	*N* (%)
Achieve target calories at the end of first week	63 (100)
Minimum protein intake >1.5 g/kg/d	63 (100)
Time to start feeding	
<48 hours	51 (81)
48–72 hours	7 (11.1)
>72 hours	5 (7.9)
Clinical feeding intolerance	
Emesis	2 (3.2)
Distension/ileus	4 (6.3)
Diarrhea	6 (9.5)
Constipation	1 (1.6)
No feeding intolerance	50 (79.4)
Complication	
Hospital-acquired infection	17 (27)
Enteral nutrition related	2 (3.2)
Parenteral nutrition related	1 (1.6)

## Data Availability

The data that support the findings of this study are available from the first author upon request.

## Supplementary Materials

Supplementary Materials Supplement 1: the enteral nutrition support protocol is used to guide enteral nutrition advancement methods and suggests management of gastrointestinal intolerance and bowel management strategies.
