# Supplementary material for: Optimal Enteral Nutrition Support Preserved Muscle Mass in Critically Ill Children
Source: J Nutr Metab. 2022 Jan 25;2022:7004543. doi: 10.1155/2022/7004543 (PMC8808230; doi:10.1155/2022/7004543)
Supplement: Supplementary Materials — Supplement 1: the enteral nutrition support protocol is used to guide enteral nutrition advancement methods and suggests management of gastrointestinal intolerance and bowel management strategies. [file 7004543.f1.pdf]

Supplement 1 The Enteral Nutrition Support Guideline

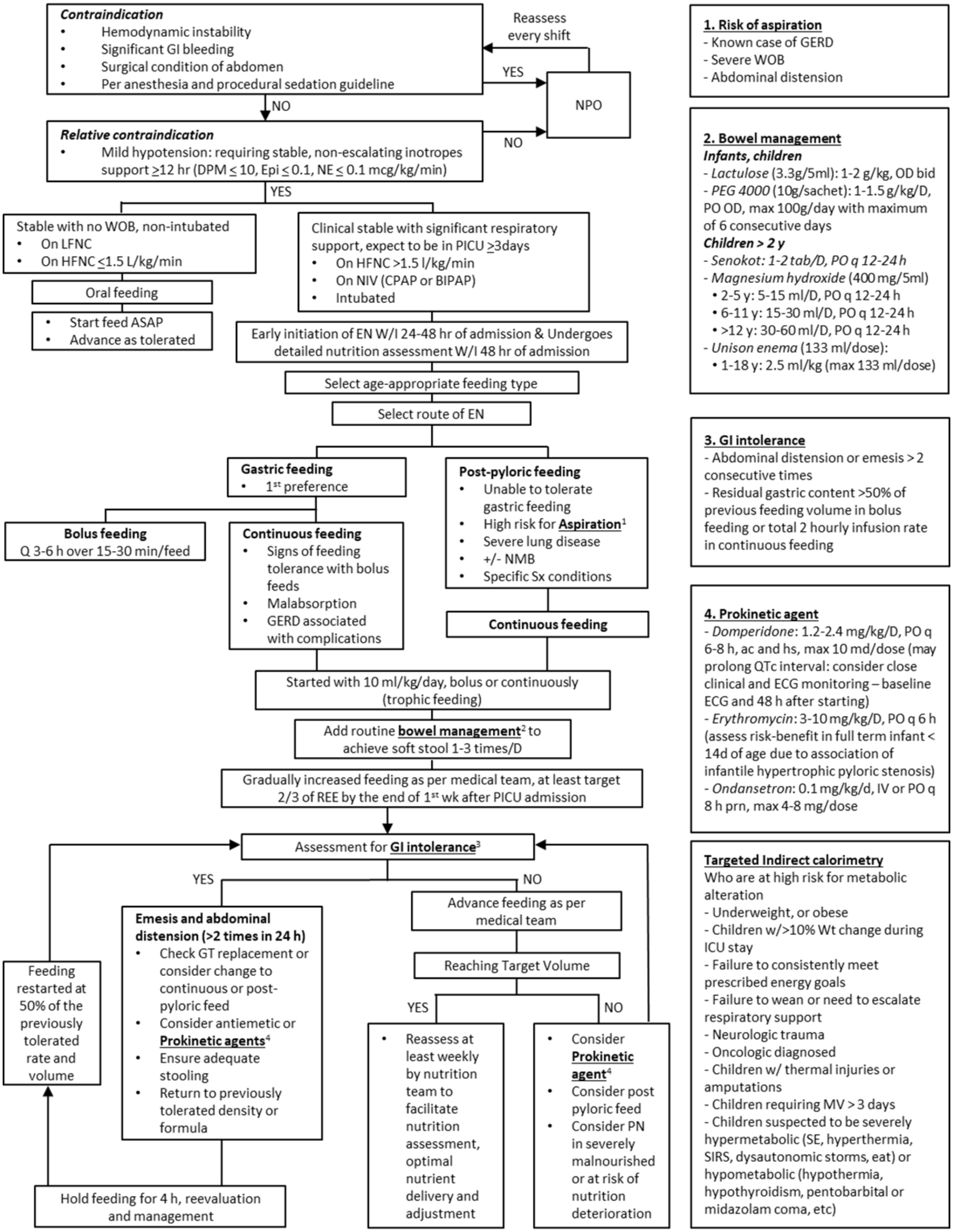

*Suggest to start PN if not reaching target energy goal by EN after first week
